# Intra-articular delivery of flurbiprofen sustained release thermogel: improved therapeutic outcome of collagenase II-induced rat knee osteoarthritis

**DOI:** 10.1080/10717544.2020.1787555

**Published:** 2020-07-05

**Authors:** Peinan Li, Haokun Li, Xiaohong Shu, Moli Wu, Jia Liu, Tangna Hao, Hongxia Cui, Lianjie Zheng

**Affiliations:** a Department of Orthopedic Surgery, Second Clinical College, Dalian Medical University, Dalian, China; b College of Pharmacy, Dalian Medical University, Dalian, China; c Department of Cell Biology, College of Basic Medical Sciences, Dalian Medical University, Dalian, China; d Department of Pharmacy, Second Clinical College, Dalian Medical University, Dalian, China

**Keywords:** Flurbiprofen, thermosensitive gel, sustained release, intra-articular drug delivery, osteoarthritis

## Abstract

Knee osteoarthritis (OA) is a common degenerative disease. Intra-articular administration of flurbiprofen is frequently employed in clinic to treat OA, while repeated injections are required because of the limited effective duration. To improve therapeutic outcome and prolong the treatment interval, a poly(*ε*-caprolactone-*co*-lactide)-*b*-poly(ethylene glycol)-*b*-poly(*ε*-caprolactone-*co*-lactide) (PCLA–PEG–PCLA) triblock copolymer based flurbiprofen thermosensitive gel for the sustained intra-articular drug delivery was designed in this study. The anti-OA effects of this flurbiprofen thermogel were investigated on collagenase II-induced rat knee OA model by multiple approaches and compared with that of conventional sodium hyaluronate and flurbiprofen injecta. *In vitro* drug release studies indicated that flurbiprofen was sustained released from the thermosensitive gel for more than three weeks. This sustained drug release system exerted comparable short-term analgesic effects and distinctly improved long-term analgesic efficacy in terms of the increased percentage of the total ipsilateral paw print intensity and the reduced Knee-Bend scores of OA rats. The inflammatory response was attenuated in the samples of flurbiprofen gel treated group by showing decreased IL-1, IL-6, and IL-11 levels in the joint fluid and down-regulated IL-1, IL-6, IL-11, COX-2, TNF-α, and NF-κB/p65 expression in the articular cartilages. The results suggest the suitability of thermosensitive copolymer PCLA–PEG–PCLA for sustained intra-articular effects of flurbiprofen and provide *in vivo* experimental evidence for potential clinical application of this flurbiprofen delivery system to better management of OA cases.

## Introduction

Knee osteoarthritis (OA) commonly occurs among middle and old populations as a kind of degenerative diseases (Jamshidi et al., 2019). OA is characterized by knee joint dysfunction and persistent pain due to joint destruction and deformation (Eitner et al., 2017). Nowadays, OA has no less impact on health and quality of life than the risk of heart attack (Törmälehto et al., 2018; Veronese et al., 2018). It has been advocated that OA should be treated as early as possible to relieve inflammatory response and to delay joint degeneration (Fan et al., 2018; Törmälehto et al., 2018). Non-pharmacological therapies such as weight loss and appropriate exercise are widespread but differ per joint (Kou et al., 2019). Pharmacological therapies are necessary depending on disease conditions. Current medication treatment is mainly based on oral drug administration, as often non-steroidal anti-inflammatory drugs (NSAIDs) (Jackson et al., 2017). Nevertheless, long-term systemic NSAIDs administration causes adverse reactions including gastrointestinal tract ulcer/bleeding, kidney/liver damage, and cardiovascular dysfunction (Scarpignato et al., 2015). Besides, oral administration of NSAIDs is associated with limited local bioavailability, and high daily dosing is needed in order to achieve therapeutic concentrations in the joint cavity, which in turn increases the adverse reactions (Yang et al., 2014; Maniar et al., 2018). The intra-articular drug injection gives the chance to enhance the safety and efficacy of OA therapy, by which drugs are directly delivered to joint cavity to perform the maximum local action and the minimum systemic exposure. As such, intra-articular administration of NSAIDs provides a reasonable choice when oral administration achieves limited local therapeutic concentrations or exhibits apparent systemic adverse reactions.

Several drugs have been already applied via intra-articular injection in OA treatment, of which flurbiprofen axetil and sodium hyaluronate (HA) are commonly used (Liu et al., 2017; Maniar et al., 2018). Flurbiprofen is an NSAID, which plays an active anti-inflammatory role by inhibiting inflammatory cytokines such as IL-6 and IL-11 (Schmitz et al., 2014; Liu et al., 2017). HA, as one of the main components of synovial fluid and cartilage matrix, plays a role of lubrication in the joint cavity to protect articular cartilage, improve joint contracture and inhibit cartilage degeneration (Liao et al., 2005; Maniar et al., 2018). Intra-articular injection of those agents can achieve better therapeutic effects in comparison with other administration routes (Bhadra et al., 2017). Even though, intra-articular injection is still a kind of invasive therapies and rapid intra-articular drug washout is associated with the need of repeated intra-articular injections, which are not patient friendly and bring about a potential risk of infection. It is therefore necessary to develop an effective way to prolong intra-articular drug retention time and cut down the frequencies of drug administration.

Recently, thermosensitive gels have attracted lots of attention as the medicinal materials. Thermosensitive gels are a kind of physical hydrogels, which could form gels spontaneously under physiological conditions thus avoiding any *in vivo* reactions or extra additives. As the injectable drug carriers, intra-articular injection of these materials could exert minimal invasiveness (Zhang et al., 2011). For the good biodegradability and biocompatibility, together with the relatively long persistence *in vivo*, the poly(*ε*-caprolactone-*co*-lactide)-*b*-poly(ethylene glycol)-*b*-poly(*ε*-caprolactone-*co*-lactide) (PCLA–PEG–PCLA) triblock copolymer based thermosensitive gel has been taken into consideration for the potential application in many medicinal fields (Zhang et al., 2011; Petit et al., 2014). This copolymer system presents a flowable sol at room temperature to enable its injectability, and transforms rapidly into an immobile gel at body temperature to form drug depots as the sustained release matrix for intra-articular drug administration (Wang et al., 2016). Drug release characteristics from this copolymer system are likely tunable by changing multiple factors, such as the injection volume, the polymer/drug concentration, and the capping group of hydroxyl ends of the copolymer, which facilitate the flexibility and feasibility as the carrier for sustained intra-articular drug delivery (Wang et al., 2016; van Midwoud et al., 2018).

In the present study, a PCLA–PEG–PCLA triblock copolymer based flurbiprofen thermosensitive gel for the sustained intra-articular drug delivery was designed. Its therapeutic efficacy and biological effects were evaluated by the use of collagenase II-induced rat knee OA model from multiple aspects and compared with that of conventional HA and flurbiprofen injecta. The underlying mechanisms of sustained anti-OA effects were also discussed. It is a novel attempt of flurbiprofen formulation and provides us with the corresponding outcome parameters for OA therapy. Unlike most published researches, particular attention is paid to the multiple evaluation of *in vivo* therapeutic efficacy (both functionally and biologically), which is indicative in the development of sustained intra-articular drug delivery systems.

## Materials and methods

### Materials

Polyethylene glycol (PEG, MW 1500), dl-lactide (LA), and *ε*-caprolactone (CL) were purchased from China National Medicines Co., Ltd. (Shanghai, China). Stannous octoate (Sn(Oct)_2_) and all other chemicals were obtained from Sigma-Aldrich (St. Louis, MO).

### Synthesis and characterization of copolymer

**Figure 1.**
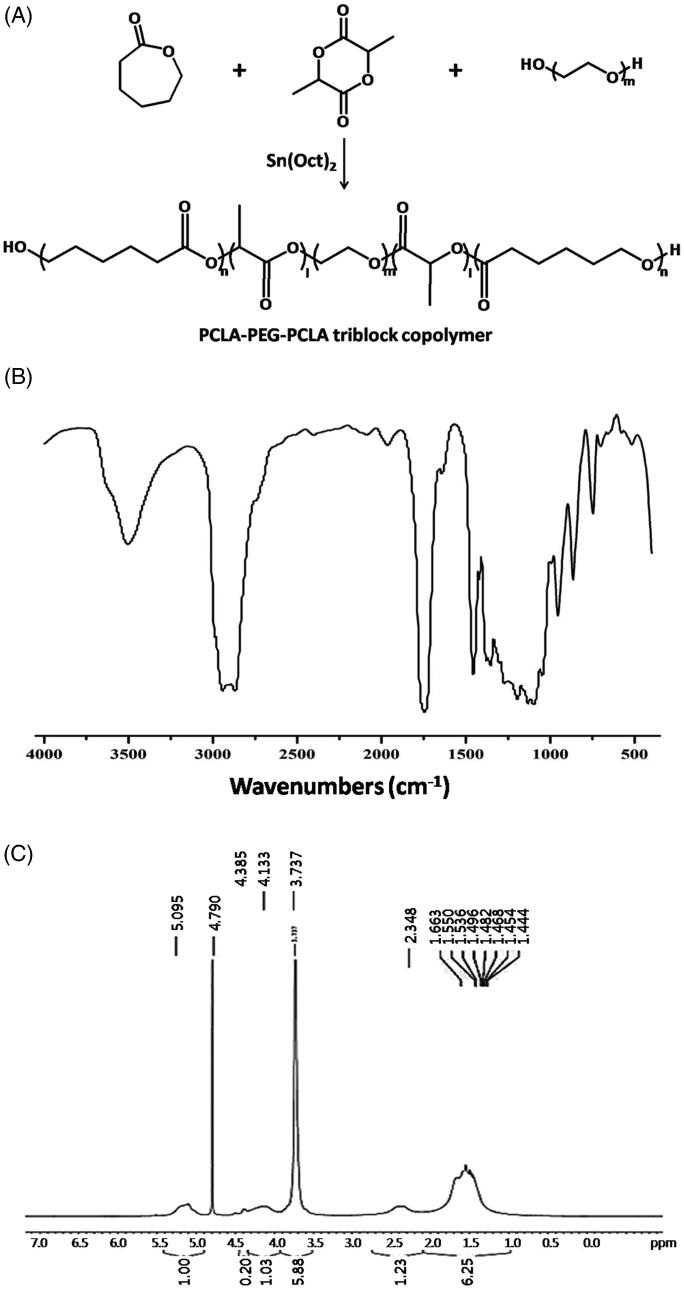
Synthesis and characterization of PCLA–PEG–PCLA triblock copolymer. The synthesis scheme (A), FTIR (B), and ^1^H NMR spectra (C).

PCLA–PEG–PCLA triblock copolymer was synthesized by ring-opening polymerization as shown in Figure 1(A) (Zhang et al., 2011). Briefly, PEG, LA, CL, and catalyst Sn(Oct)_2_ (1% by weight of reactant) were weighed at the ratio of 0.9:1:1 and introduced into a dry polymer tube. The reaction was carried out at 150 °C for eight hours under nitrogen atmosphere and then cooled to room temperature. The product was purified by dissolved in water (<10 °C) and heated to 70–80 °C to precipitate the copolymer. The resulting copolymer PCLA–PEG–PCLA was dried under vacuum at room temperature for 48 hours, and characterized by FTIR and ^1^H NMR spectroscopy.

### Preparation of flurbiprofen thermosensitive gel

Flurbiprofen thermosensitive gel was prepared by thin film dispersion method (Petit et al., 2014). One gram of flurbiprofen was dissolved in acetone with 15, 20, and 25 g of the PCLA–PEG–PCLA copolymer, respectively. The solution was incubated in the water bath at 40 °C, and the solvent was then removed by rotary evaporation. A thin film was formed on the wall of the test tube, and a suitable amount of distilled water was added at room temperature to dissolve the mixture and make the final volume to 100 mL. The resulting thermosensitive gels contained 1% (w/v) of flurbiprofen combined with 15%, 20%, or 25% (w/v) of the PCLA–PEG–PCLA copolymer. The sol–gel phase transition was checked at 25 °C and 37 °C, respectively (Petit et al., 2014).

### *In vitro* drug release studies

*In vitro* drug release studies were performed according to the methods reported in the references (Zhang et al., 2011; van Midwoud et al., 2018). The thermosensitive gel (1 mL) was placed into the test tube and incubated at 37 °C in water bath. When the gel phase was completely formed, 8 mL of release medium (pH 7.4 phosphate-buffered saline containing 0.02% (w/v) NaN_3_) was added. The medium was maintained at 37 °C with constant stirring at a speed of 50 rpm. The effective release area was 1.0 cm^2^. At predetermined time intervals, the release medium was all removed for flurbiprofen measurement and the same volume of fresh release medium was added immediately to ensure the sink condition. Samples were filtered by 0.45 μm microporous membrane and measured by the high-performance liquid chromatography (HPLC). The drug release curve was obtained by plotting the cumulative drug release amount against the sampling time.

### Establishment of rat OA model

Forty-five Sprague-Dawley rats (male, 24 week old) were obtained from the Animal Center of Dalian Medical University. The rats were housed in polycarbonate cages with hard wood chips at a temperature of 23–25 °C and a humidity of 55–65% with a 12-hour light/dark cycle. Diet and drinking water were available *ad libitum*. After a one-week acclimation period, the animals were subjected to OA induction by injecting 25 μL collagenase II (500 U/mL; Sigma-Aldrich, St. Louis, MO) into the right knee joint under general anesthesia for two times in three-day interval (Takayama et al., 2014).

### Experimental groups and intra-articular administration

Six weeks after the second injection of collagenase II into the rats' articular cavity, the 45 rats were randomly divided into five groups (nine rats/group): (1) the blank gel group, intra-articular injection of thermosensitive gel with no flurbiprofen loaded (50 μL/time); (2) the flurbiprofen group, intra-articular injection of flurbiprofen injecta (0.5 mg/time); (3) the flurbiprofen gel group, intra-articular injection of flurbiprofen thermosensitive gel (0.5 mg/50 μL/time); (4) the HA group, intra-articular injection of sodium HA injecta (0.5 mg/50 μL/time); (5) the untreated group, OA rats without any treatment as control. Intra-articular drug administration was conducted in 72-hour intervals for seven times (Takayama et al., 2014). The animal experiments were performed under chloral hydrate anesthesia and all efforts were made to minimize suffering.

### Pain degree estimation

The rats were estimated in regular intervals of each ankle joint for responses to movement by the use of CatWalk and Knee-Bend tests (Adães et al., 2015; Sagar et al., 2015). In the CatWalk test, both hind paws of the tested rat were put on the inkpad and then the rat was allowed to walk freely on a piece of white paper to record the paw prints. The paw prints were transferred to digital images by scanning, and the pixel values and paw areas were calculated according to the method described in our previous studies (Li et al., 2012). The results were expressed as the percentage of the total ipsilateral paw print intensity (%TIPPI) in the sum of both paw prints. The Knee-Bend test was conducted after the CatWalk one. Briefly, the scales of the vocalization and struggle reactions in response to five repeats of knee joint flexions and extensions were scored as: 0, no response to joint flexions/extensions; 0.5, struggle to maximal flexions/extensions; 1, struggle to moderate flexions/extensions with vocalizations to maximal flexions/extensions; 2, vocalizations to moderate flexions/extensions. The scores of individual experimental groups were summarized. The corresponding data of the contralateral healthy knee were collected first for comparing the difference between the ipsilateral and the contralateral Knee-Bend score.

### Sample collection

Joint fluid collection was conducted before the first and 72 hours after the last treatment. In order to minimize animal suffering and the influence of result evaluation, 5 μL joint fluid was extracted from the individual articular cavities. The samples collected from the same experimental group were pooled and subjected to centrifugation in 3000 rpm. The supernatant was used for ELISA determination of inflammatory factors, and the sediment was used for white blood cell counting and fractionation. Seventy-two hours after the last treatment, the animals were killed and the articular cartilage of the knee joint was exposed and sampled for histological examination and protein preparation according to our previous reports (Li et al., 2015).

### Peripheral blood picture examination

Caudal vein blood samples were collected from the rats before and after the flurbiprofen administration, and subjected to conventional treatment for blood picture examination (He et al., 2017). The tests included the determination of hemoglobin concentration and white blood cell differential count (WBC-DC).

### ELISA-based cytokine detection in joint fluids

As the amount (5 μL) of joint fluid collected from each of the rats was not sufficient for multi-parameter examination, the nine joint fluid samples from the same group were mixed and analyzed together. The results obtained were regarded as the mean values. The ELISA assay was conducted to determine serum IL-1, IL-6, and IL-11 levels of the experimental groups according to the supplier's instruction (Bioswamp Biosci. Inc., Shanghai, China).

### Protein extraction and Western blotting

For protein preparation, the frozen knee articular cartilage tissues in the same group were sectioned to 5-μm slices (four slices/sample) and gathered together by the method described in our earlier studies (Li et al., 2015). The sample proteins (20 μg/well) were separated in 10% sodium dodecylsulfate-polyacrylamide gel electrophoresis and transferred to polyvinylidene difluoride membrane (Roche GmbH, Mannheim, Germany). The membrane was blocked with 5% skimmed milk in TBS-T (10 mM Tris–HCl, pH 8.0, 150 mM NaCl and 0.5% Tween 20) at 4 °C, rinsed 10 minutes for three times with TBS-T, followed by three-hour incubation at room temperature with the first antibody and then one-hour incubation with HRP-conjugated anti-mouse or anti-rabbit IgG (Zymed Lab., Inc., South San Francisco, CA). The bound antibody was detected using the enhanced chemiluminescence system (Roche GmbH, Mannheim, Germany). After removing the labeling signal by incubation with stripping buffer, the membrane was reprobed with other antibodies one by one until all of the parameters were examined. The rabbit anti-rat antibodies against IL-1, IL-6, IL-11, COX-2, and NF-κB (Zymed Lab., Inc., South San Francisco, CA) were used in the analyses. β-Actin was cited as quantitative control.

### Statistical analysis

The experimental data were analyzed by SPSS 13.0 statistical software (SPSS Inc., Chicago, IL) and presented as mean ± standard deviation. The differences in continuous variables were analyzed by Student's *t*-test or one-way ANOVA. Statistical significance was defined as *p* < .05.

## Results

### Characterization of PCLA–PEG–PCLA triblock copolymer

The PCLA–PEG–PCLA triblock copolymer was synthesized by ring-opening polymerization of CL and LA using PEG as initiator and Sn(Oct)_2_ as catalyst. The synthesis scheme is schematically illustrated in Figure 1(A), and the chemical structure was confirmed by FTIR and ^1^H NMR. In FTIR spectra (Figure 1(B)), the peak at 3500 cm^−1^ was assigned to the terminal hydroxyl groups of PCLA–PEG–PCLA. The bands at 2937 cm^−1^ and 2870 cm^−1^ were attributed to the C–H stretching vibration. The strong absorption at 1750 cm^−1^ was due to the stretching vibration of the C═O group. In ^1^H NMR spectra (Figure 1(C)), the characteristic signals were observed as follows. The peaks at 5.10 ppm were attributed to methine protons of –COC***H***(CH_3_)O– in the lactide units. The peaks at 3.74 ppm were assigned to methylene protons of –C***H_2_***C***H_2_***O– in the ethylene glycol units. The peaks at 2.35 ppm were due to methylene protons of –COC***H_2_***CH_2_CH_2_CH_2_CH_2_O– in the caprolactone units (Zhang et al., 2011).

### Phase transition and *in vitro* drug release of thermosensitive gel

**Figure 2.**
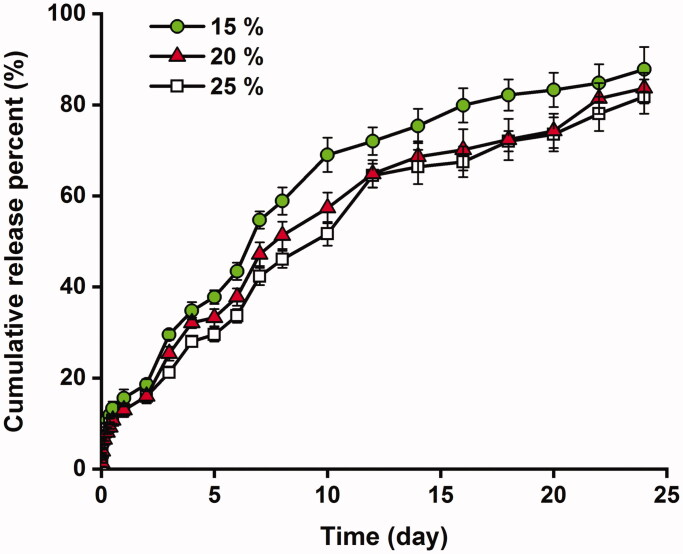
*In vitro* release profiles of flurbiprofen from the thermosensitive gels. The drug loading was 1% (w/v); the copolymer concentration was 15%, 20%, and 25% (w/v).

The sol–gel phase transition of thermosensitive gel was ascertained at 25 °C and 37 °C. The copolymer system presented an injectable sol at room temperature and an immobile gel after heated to the body temperature, which was consistent as reported (Zhang et al., 2011). To be specific, as the copolymer concentration increased from 15% to 25%, the gelling temperature decreased from 31 °C to 30 °C and 28 °C, respectively. It was in accordance with the reports and a result of the increased hydrophobic interaction (Qiao et al., 2005). The sol-to-gel conversion enabled the potential of the PCLA–PEG–PCLA copolymer based thermosensitive gel as an injectable material for sustained release of flurbiprofen in medical applications. The *in vitro* release profiles of flurbiprofen from thermosensitive gels are shown in Figure 2. As indicated, a sustained drug release lasting more than three weeks was achieved without initial bursts. The cumulative drug release from the hydrogel was about 80% on a 24-day observation. An increase in the copolymer concentration from 15% to 25% (w/v) modified the release rate of flurbiprofen, resulting in a relatively steady drug release rate throughout the *in vitro* observation. Accordingly, flurbiprofen thermosensitive gel containing 25% (w/v) of the PCLA–PEG–PCLA triblock copolymer was used in the subsequent experiments to evaluate the therapeutic outcome.

### Flurbiprofen gel exerted comparable short-term analgesic effects

**Figure 3.**
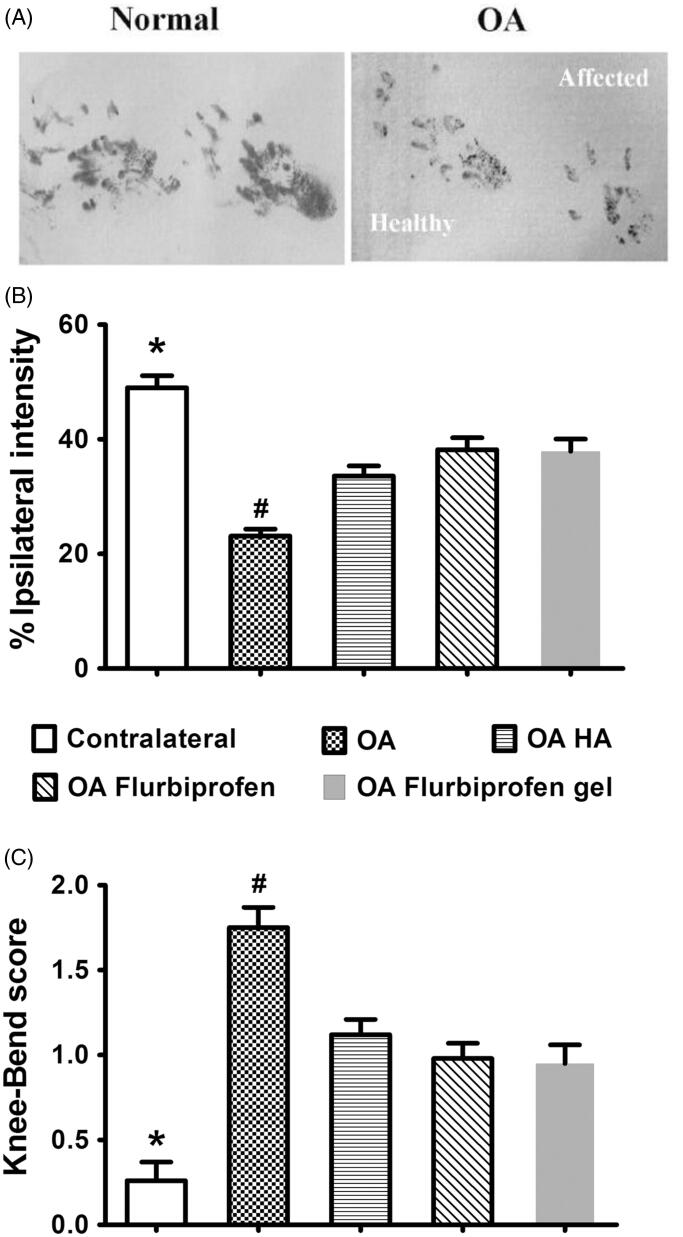
Comparable short-term analgesic effects of flurbiprofen thermogel on OA rats. (A) The CatWalk test based on the intensities of the paw prints of the contralateral healthy sides (Healthy) and the ipsilateral affected OA sides (Affected). %TIPPI (B) and the Knee-Bend scores (C) of the four experimental groups before and 2 hours after the first drug treatments. The data collected from the contralateral sides of the individual rats are cited as the normal control. *p < .01 in comparison with the data of other four experimental groups; ^#^*p* < .05 in comparison with the data of HA, flurbiprofen, and flurbiprofen gel groups. No statistical difference of the data from the three drug treated groups.

On basis of the intensities of the paw prints (Figure 3(A)), the CatWalk test was used to evaluate the efficacy on improving rat movement disability caused by OA. Before the first drug treatments, the original %TIPPI in the four groups was 23.1 ± 1.24 in comparison with 49.0 ± 2.10 of the contralateral counterparts. Two hours after the treatments, the percentages were increased to 33.6 ± 1.76 in HA group, 38.2 ± 2.07 in flurbiprofen group, and 37.9 ± 2.14 in flurbiprofen gel group (Figure 3(B)). No statistical difference was established between the data in flurbiprofen gel group and those in HA and flurbiprofen groups (*p* > .05). The result of the Knee-Bend test was in accordance with that of the CatWalk one in terms of the reduced response scores from 1.75 ± 0.12 to 1.12 ± 0.09 in HA group, to 0.98 ± 0.09 in flurbiprofen group and to 0.95 ± 0.11 in flurbiprofen gel group (Figure 3(C)).

### Flurbiprofen gel exerted better sustained analgesic effects

**Figure 4.**
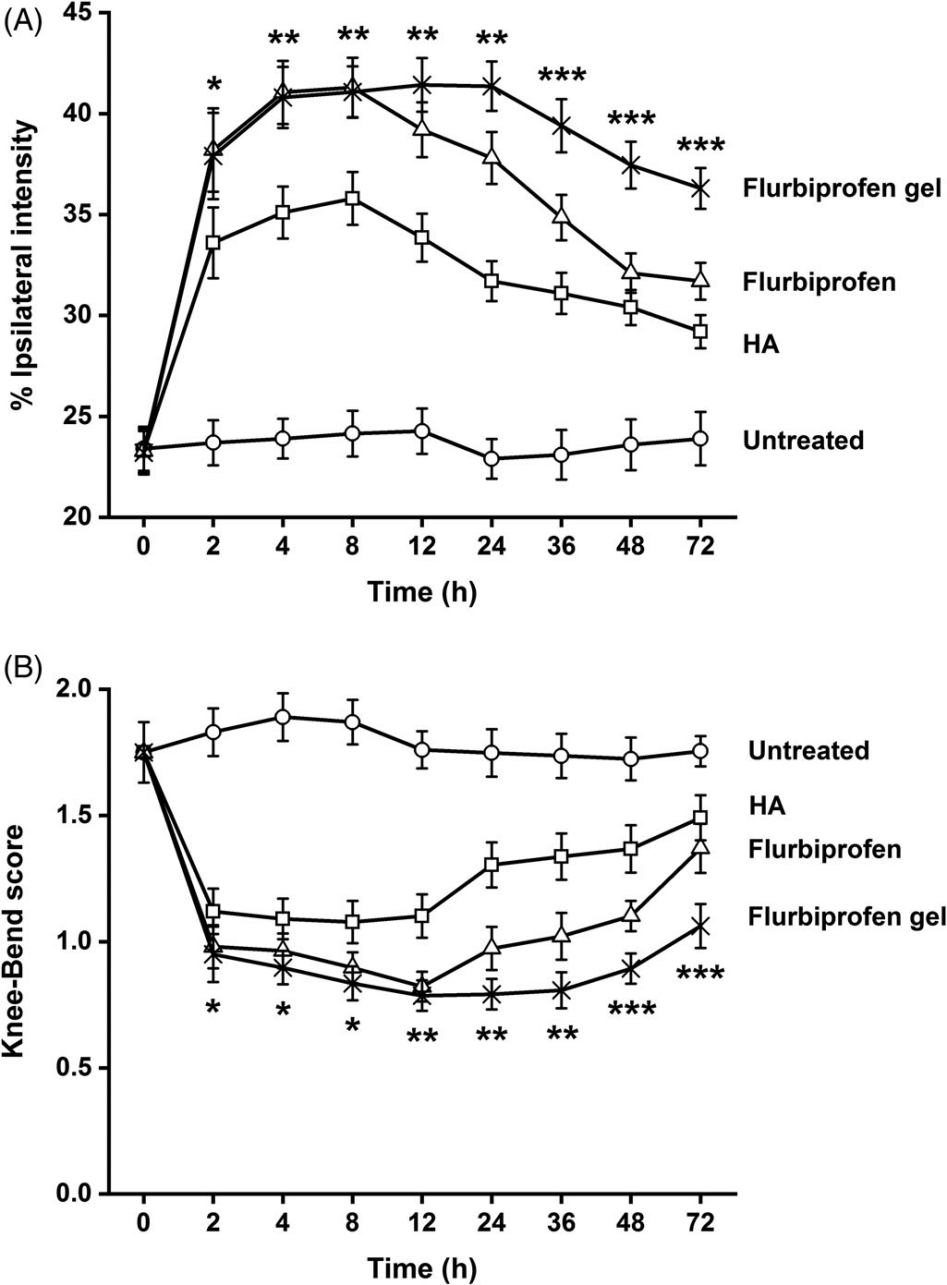
Better sustained analgesic effects of flurbiprofen thermogel. The sequential Knee-Bend (A) and CatWalk (B) tests performed on the rats in each experimental group at the time points of 0, 2, 4, 8, 12, 24, 36, 48, and 72 hour after the first-time drug treatments. Significant differences of the final scores are established between flurbiprofen gel group and the other experimental groups (*p* < .05). *p < .01 between the flurbiprofen gel group and the untreated group; **p < .05 between the flurbiprofen gel group and the HA group; ***p < .05 between the flurbiprofen gel group and the flurbiprofen group.

The sequential CatWalk and Knee-Bend tests were conducted on the rats in each experimental group at the time points of 0, 2, 4, 8, 12, 24, 36, 48, and 72 hours after the first-time drug treatments. As shown in Figure 4(A), the %TIPPI of HA and flurbiprofen groups increased to the peak values (35.8 and 41.3) at eight hour point and then gradually decreased in time-related patterns to 29.2 and 31.7 at 72 hour point. In the Knee-Bend test, the rats in HA and flurbiprofen groups showed similar responses to knee joint flexions and extensions from 2 hour to 8 hour points and increased the response scales thereafter (Figure 4(B)). The %TIPPI and the response scores of flurbiprofen gel group remained relatively stable throughout the examination, which reached to 36.3 and 1.06 at 72 hour point. Significant differences of the final scores were established between flurbiprofen gel group and the other experimental groups (*p* < .05).

### Flurbiprofen gel effectively improved OA symptoms

**Figure 5.**
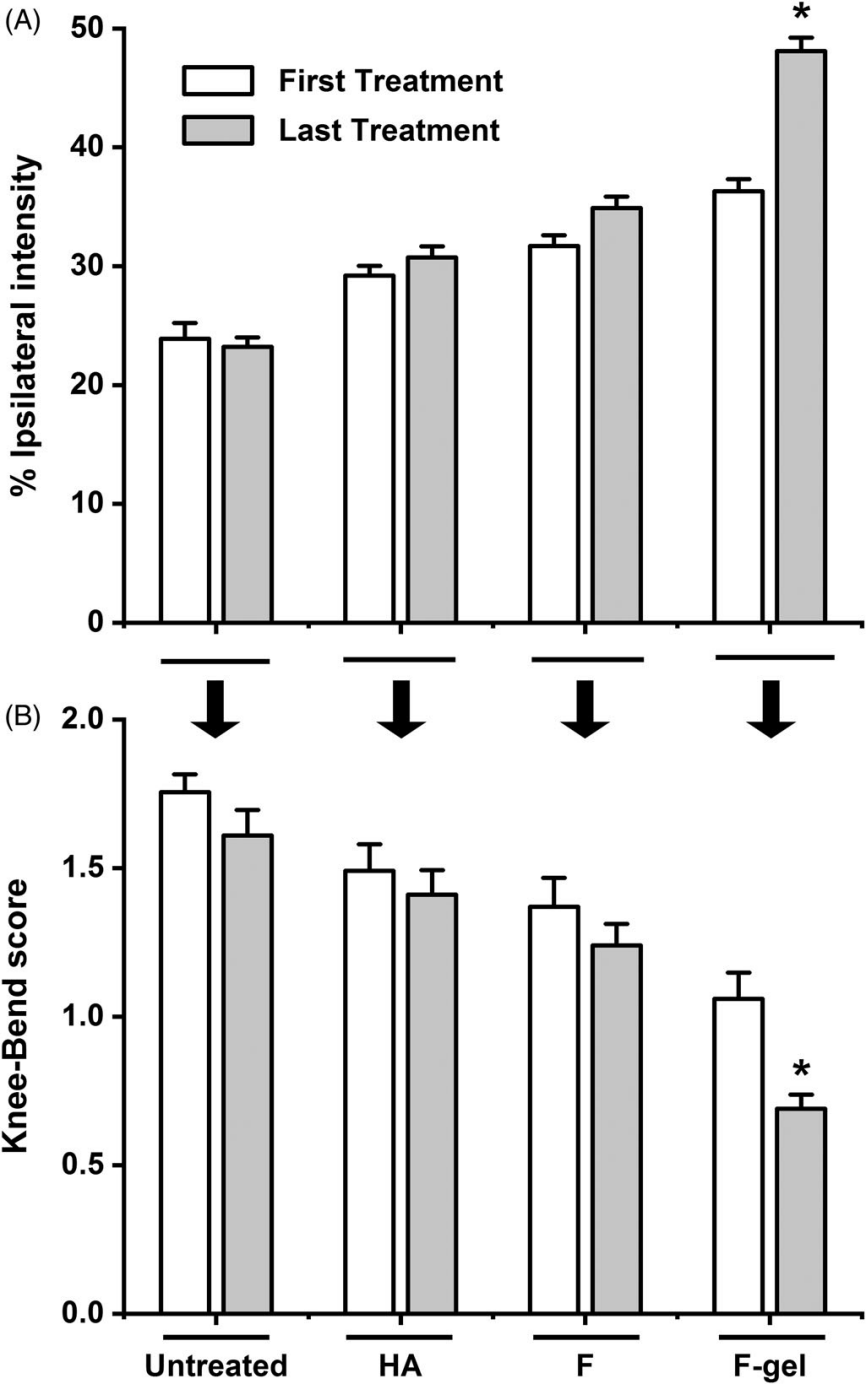
Flurbiprofen gel effectively improved OA symptoms. Measurement of the %TIPPI (A) and the Knee-Bend scores (B) of the experimental groups 72 hours after the first and the last treatments. Untreated, OA rats without treatment; HA, with sodium hyaluronate treatment; F, with flurbiprofen treatment; F-gel, with flurbiprofen gel treatment. *The first and the last %TIPPI and Knee-Bend score of flurbiprofen gel group are significantly different (*p* < .01). The differences of paired parameters of other groups are not distinct (*p* > .05).

The %TIPPI and the Knee-Bend response scores of the experimental groups were measured 72 hours after the final drug treatments and compared with those obtained from the same group 72 hours after the first drug treatments. It was found that the last %TIPPI of HA, flurbiprofen, and flurbiprofen gel groups were increased in the extents of 5.2% (from 29.2 to 30.7), 10.1% (from 31.7 to 34.9), and 32.6% (from 36.3 to 48.1), respectively (Figure 5(A)). The following Knee-Bend test revealed that the response scores were reduced from 1.49 to 1.41 (5.4%) in HA group, from 1.37 to 1.24 (9.5%) in flurbiprofen group, and from 1.06 to 0.69 (34.9%) in flurbiprofen gel group (Figure 5(B)). The last %TIPPI and Knee-Bend score of flurbiprofen gel group were significantly different from those of HA and flurbiprofen groups (*p* < .01) as well as the ones after the first flurbiprofen gel treatment (*p* < .01).

### No influence of flurbiprofen gel in rat blood picture

To elucidate the potential influence of the flurbiprofen gel on the rat health, the hemoglobin concentration, the peripheral total white blood cell number, differential count, and coagulation profile were analyzed before and after the treatments of flurbiprofen and its thermosensitive gel form. The results revealed no significant change (*p* > .05) of the blood parameters between the samples collected before and after the treatments (data shown in Supplementary Table), indicating the limited adverse effect of long-term intra-articular administration of the two flurbiprofen reagents on the general condition of rats.

### Flurbiprofen gel decreased IL-1, IL-6, and IL-11 levels in joint fluid

**Figure 6.**
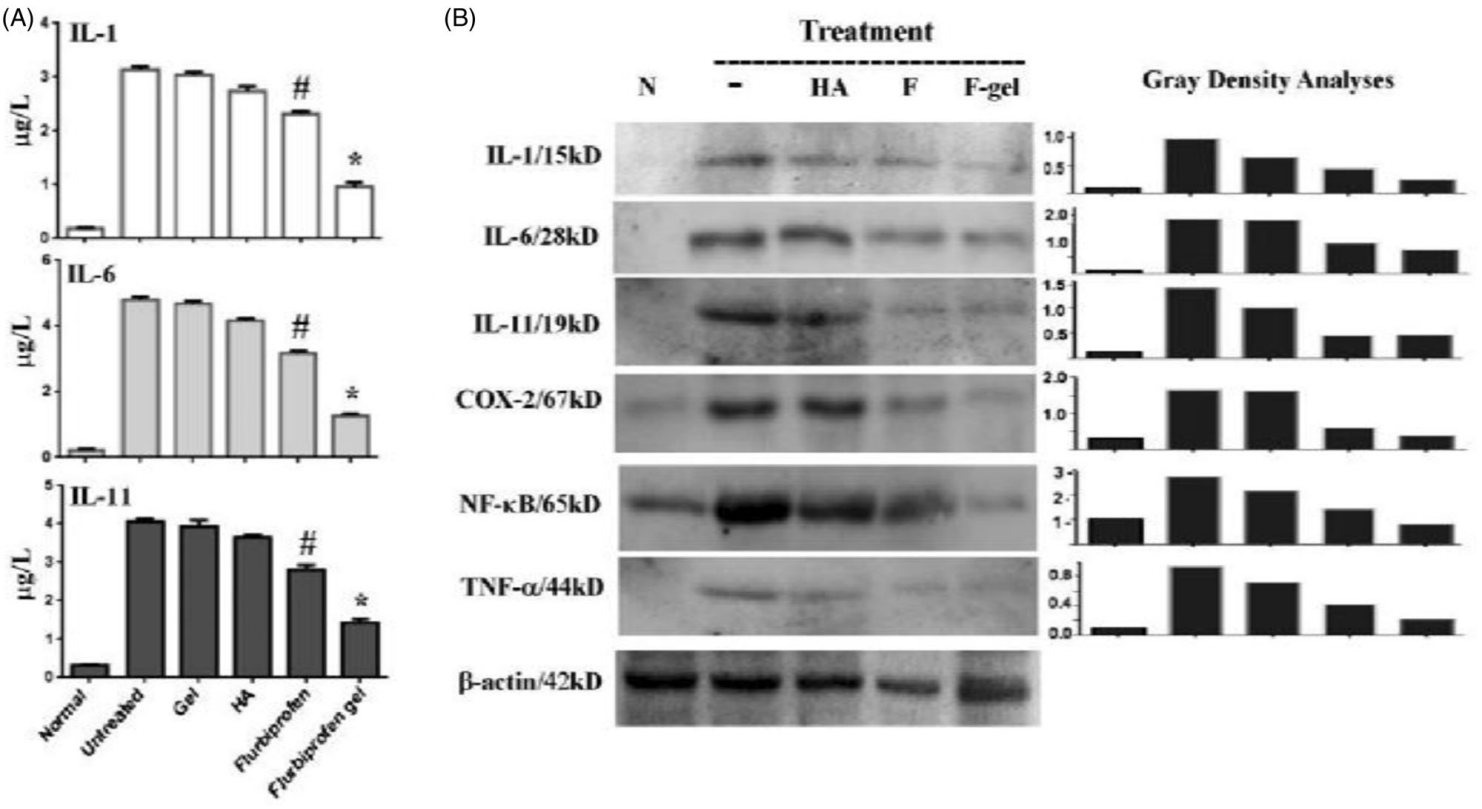
Flurbiprofen gel suppressed local inflammatory response of OA rats. (A) IL-1, IL-6, and IL-11 oriented ELISA assay performed on the pooled joint fluids collected from the normal rats (Normal), the OA rats without treatment (Untreated) and the OA rats 72 hours after the last drug-free gel (Gel), HA, flurbiprofen, or flurbiprofen gel treatment. ^#^*p* < .05 in comparison with the data of normal, untreated, gel, and HA groups; *p < .01 in comparison with the data of other experimental groups. (B) Western blotting demonstration and gray density quantification of IL-1, IL-6, IL-11, COX-2, TNF-α, and NF-κB/p65 expression patterns in the articular cartilages of normal rats (N) and OA rats without treatment (–) or treated by intra-articular injection of HA (HA), flurbiprofen (F), or flurbiprofen gel (F-gel). The samples were collected 72 hours after the last treatment.

As shown in Figure 6(A), the ELISA assay performed on the joint fluids revealed that IL-1, IL-6, and IL-11 levels in the normal joint samples were 0.192 μg/L, 0.212 μg/L, and 0.327 μg/L, which were increased to 3.13 μg/L, 4.75 μg/L, and 4.06 μg/L before the treatments. Blank thermosensitive gel by itself decreased their levels without significant difference (*p* > .05). Flurbiprofen reduced IL-1, IL-6, and IL-11 levels to 2.31 μg/L, 3.15 μg/L, and 2.86 μg/L significantly (*p* < .05) and HA reduced IL-1, IL-6, and IL-11 levels to 2.78 μg/L, 4.12 μg/L, and 3.69 μg/L, respectively. The levels of IL-1, IL-6, and IL-11 in the flurbiprofen gel group were 0.93 μg/L, 1.27 μg/L, and 1.44 μg/L with significant difference in comparison with other experimental groups (*p* < .01).

### Flurbiprofen gel down regulated inflammatory factor expression

As IL-1, IL-6, IL-11, COX-2, TNF-α, and NF-κB/p65 are known as OA promoting factors (Kim et al., 2018; Tachmazidou et al., 2019), their expression patterns in the articular cartilages of the experimental groups were examined at the end of treatments via Western blotting. Samples were isolated from the pooled articular cartilages of the individual groups. Samples prepared from the articular cartilages of the contralateral sides of the rats were cited as normal control. The results of Western blotting (Figure 6(B)) revealed that IL-1, IL-6, IL-11, COX-2, TNF-α, and NF-κB/p65 were expressed in low levels in the healthy articular cartilages. Those proteins were remarkably upregulated in the untreated OA groups and were decreased in the samples of flurbiprofen and HA groups. More distinct reductions of IL-1, IL-6, COX-2, TNF-α, and NF-κB/p65 were found in flurbiprofen gel treated samples in comparison with the untreated, HA-treated as well as flurbiprofen-treated ones.

## Discussion

Intra-articular drug injection has long been used to treat OA (Adães et al., 2014; Takayama et al., 2014), and the development of new analgesic and anti-inflammatory drugs further improves its therapeutic outcome (Martel-Pelletier et al., 2016). However, the duration of drug effects is quite limited because of the quick elimination of drugs from the joint cavity (Joshi et al., 2018). It would be therefore of clinical values to explore reliable ways to prolong intra-articular drug retention time for better management of OA. It has been found that some medicinal polymers are the ideal excipients for the sustained drug delivery, of which the temperature sensitive polymers such as PCLA–PEG–PCLA triblock copolymer have been applied to the drug injection system (Zhang et al., 2011; Bajpayee et al., 2017). PCLA–PEG–PCLA, as an injectable biodegradable material, has been reported to have good biocompatibility with little cytotoxicity and hemolysis, and mild inflammatory response. Therefore, PCLA–PEG–PCLA copolymer was synthesized and employed as a carrier to prepare the sustained release gel for intra-articular administration. Before the formal experiment, the potential influence of the continuous injection of the no-load thermosensitive gel on the animals' general state and blood picture as well as the possible local stimulation was analyzed first. The results showed that the parameters checked remained almost unchanged after seven times (in 72-hour intervals) of injection. In addition, there was no stimulatory adverse effect on the joints in terms of low levels of inflammatory factors in articular fluid and unchanged morphological feature of articular cartilage. The safety of this sustained release material is further ascertained and, therefore, suitable to our research purposes.

As is generally known, pharmaceutical preparations with better drug release characteristics may increase the safety and efficacy of drugs as well as the compliance of patients. *In vitro* drug release studies demonstrated that an increase in the copolymer concentration in the hydrogel from 15% to 25% (w/v) modified the release of flurbiprofen, resulting in a relatively steady drug release rate throughout the *in vitro* observation. PCLA–PEG–PCLA is a triblock copolymer composed of hydrophilic PEG block and hydrophobic PCLA block. Flurbiprofen belongs to a propionic acid class of NSAIDs. The carboxyl group and the fluorine atom in the structure as proton donors and acceptors could interact with the copolymer by hydrogen bonding interaction (Qiao et al., 2005; Cui et al., 2016). Thus, an increase in the copolymer concentration in the hydrogel increased the interaction with the drug molecular, which contributed to a relatively steady drug release rate (Gao et al., 2011; Cui et al., 2015). Though efforts have been made to mimic the *in vivo* situation, we should notice that differences still exist between *in vitro* and *in vivo*. In fact, the situation *in vivo* is more complicated, which is associated with enzyme-assisted degradation, continuous solute exchange by *in vivo* joint microcirculation, etc. (Yang et al., 2014). In some cases, the retention time of a sustained delivery system *in vivo* were reported much shorter than that *in vitro* (Sandker et al., 2013; Petit et al., 2014; Tellegen et al., 2018). Other associated results were faster release rates or shorter lag time (Petit et al., 2014; van Midwoud et al., 2018). Therefore, a series of *in vivo* studies were conducted in the present study to further evaluate the local therapeutic efficacy of the sustained intra-articular drug delivery system, which is worthwhile and more informative.

Currently, the NSAIDs and HA are frequently used in clinic. Flurbiprofen is considered as one of the best NSAIDs for OA, because of its potent anti-inflammatory and analgesic effects (Sugimoto et al., 2016a). HA has been approved by the U.S. Food & Drug Administration (FDA) as an intra-articular viscosupplementation, which is of good therapeutic efficacy and excellent clinical safety (Concoff et al., 2017). Besides, due to its unique cross-linking behavior, intra-articular administration of HA has a longer-lasting benefit over other drugs, such as corticosteroids (Thomas et al., 2017). For these reasons, HA was used as a comparative material, together with flurbiprofen injecta, to evaluate the sustained effects of the flurbiprofen thermosensitive gel for OA therapy. The dose was selected according to the relevant references (Fukumoto et al., 2018; Maniar et al., 2018). The analgesic effect was elucidated using rat OA model. The analyses of %TIPPI and Knee-Bend scores revealed that the analgesic effects of flurbiprofen thermosensitive gel was similar with that of flurbiprofen injecta at two hour point but became more distinct at 72 hour point after administration. Direct intra-articular administration allows for an effective concentration where it is needed with minimum amounts of drugs (Janssen et al., 2014), and a high initial concentration at the site of action is prone to be achieved compared with systemic administration (Yang et al., 2014). Consequently, the therapeutic outcome was observed after two hours upon intra-articular injection. Similar results were reported in other researches, in which, high local concentrations of drugs were achieved in a relatively short time associated with sustained intra-articular drug delivery systems (Thakkar et al., 2007; Tellegen et al., 2018). In terms of the increased %TIPPI and the decreased Knee-Bend response scores, we therefore consider that one-time flurbiprofen gel treatment can prolong the analgesic time and the drug administration interval. It would be possible that the suffering of OA animals and patients would be assuaged.

Pain scores are referring index for predicting the efficacy of intra-articular injection, and CatWalk and Knee-Bend tests are the ones commonly used in rat OA experimental model (Adães et al., 2014, 2015; Sagar et al., 2015). These two tests were thus employed to grade the pain of flurbiprofen and HA treated rats after the first and the last drug administrations. It was found that the pain was partly released in the extents of 10.1% and 9.5% in the former and 5.2% and 5.4% in the later case, possibly due to the delayed effects of residual drugs. The better analgesic outcome of flurbiprofen treated group may result from its anti-inflammatory effect(s). Interestingly, the final average %TIPPI of the flurbiprofen gel group was distinctly increased and the Knee-Bend score was distinctly decreased in comparison with the corresponding parameters collected 72 hours after the first treatment. These findings suggest that flurbiprofen gel can relieve pain more efficiently. Moreover, flurbiprofen gel greatly increased the final %TIPPI and decreased Knee-Bend score in comparison with flurbiprofen form. These results indicate that flurbiprofen in the thermosensitive gel can maintain its analgesic and anti-inflammatory activities through sustained drug release. As inflammation is considered to be the main cause of joint pain and certain subjective factors may affect the accuracy of the data collected from CatWalk and Knee-Bend tests, it would be worthwhile to further analyze the statuses of inflammation-related factors in order to ensure the improved therapeutic efficacy and to explore the underlying anti-OA mechanism of this agent.

The pain degree of OA is closely related to the severity of joint inflammation which can be determined or reflected by the levels of inflammation-related factors in the synovial fluid as well as in the chondrocytes and synovial cells (Philpott et al., 2017). It has been known that some cytokines such as IL-1, IL-6, and IL-11 contribute to the OA pathogenesis through activating proinflammatory signaling pathways such as NF-κB-mediated signal transduction (Philpott et al., 2017; Kim et al., 2018; Tachmazidou et al., 2019) and activated NF-κB signaling triggers the expression of inflammation-promoting genes including COX-2, a molecular target of flurbiprofen (Sugimoto et al., 2016b). Therefore, understanding the state of cytokines in the synovial fluid before and after treatment is an approach to assess anti-inflammatory efficacy of flurbiprofen thermosensitive gel in the molecular level. In other words, efficient reduction of inflammatory cytokines may release both inflammation and pain. Flurbiprofen has been known to have the above actions (Sugimoto et al., 2016b). Our ELISA and immunohistochemical results clearly demonstrated that the levels of inflammatory factors so far checked were lower in the flurbiprofen group than that in the HA group and the former group was accompanied with better pain remission. In the case of the flurbiprofen gel group, remarkable reduction of IL-1, -6, and -11 production, COX-2 expression and NF-κB/p65 nuclear translocation were evidenced in the synovial fluids and chondrocytes with further improved pain remission. It would be reasonable to consider that this thermosensitive gel facilitates the sustained release of flurbiprofen to continuously exert its anti-inflammatory and analgesic effects in the intra-articular cavity. These promising results explained why the pain scores of flurbiprofen gel group were lower than that of the untreated and the conventional flurbiprofen group at the first 72 hour point and, especially by the end of the experiment. Therefore, the flurbiprofen gel used here may provide an approach to improve OA treatment and, therefore, would be of potential clinical values.

## Conclusions

A PCLA–PEG–PCLA triblock copolymer based flurbiprofen thermosensitive gel for the sustained intra-articular drug delivery was designed and evaluated in this study. Intra-articular administration of this sustained drug release system effectively prolonged the analgesic time and remarkably reduced the inflammatory response of rat OA model by downregulating inflammatory factors expression. The results of the current study suggest the suitability of thermosensitive copolymer PCLA–PEG–PCLA for sustained intra-articular effects of flurbiprofen and provide an experimental basis for potential clinical application of this flurbiprofen hydrogel to better management of OA patients.

## Supplementary Material

Supplemental Material
