# Supplementary material for: Intra-articular delivery of flurbiprofen sustained release thermogel: improved therapeutic outcome of collagenase II-induced rat knee osteoarthritis
Source: Drug Deliv. 2020 Jul 5;27(1):1034–43. doi: 10.1080/10717544.2020.1787555 (PMC8216450; doi:10.1080/10717544.2020.1787555)
Supplement: Supplemental Material [file IDRD_A_1787555_SM8952.docx]

**Supporting Information**

**Supplementary Tables**

**Table.** Routine blood test performed on OA rats before and after flurbiprofen and flurbiprofen gel treatments.

| Item | Flurbiprofen* | | Flurbiprofen gel* | |
| --- | --- | --- | --- | --- |
|  | Before | After | Before | After |
| Hemoglobin concentration (g/L) | 147.71 ± 16.32 | 145.32 ± 14.67 | 147.92 ± 16.46 | 148.53 ± 15.02 |
| Total WBCs (× 10^9^/L) | 18.42 ± 6.93 | 19.81 ± 7.22 | 18.13 ± 6.71 | 17.42 ± 6.56 |
| Lymphocytes (× 10^9^/L) | 12.61 ± 5.63 | 14.26 ± 6.75 | 12.77 ± 5.42 | 12.41 ± 5.31 |
| Neutrophils (× 10^9^/L) | 5.81 ± 0.58 | 5.55 ± 0.42 | 5.36 ± 0.47 | 5.01 ± 0.37 |
| PLT (× 10^9^/L) | 693.00 ± 179.21 | 687.31 ± 173.46 | 689.00 ± 173.09 | 691.00 ± 181.21 |
| Plasma prothrombin time (Ss) | 15.31 ± 1.18 | 14.86 ± 1.08 | 14.98 ± 1.12 | 15.37 ± 1.26 |

*, No statistical difference between the parameters before and after drug treatment (*P* > 0.05).
